# Investigating the relationship between vitamin-D deficiency and glycemia status and lipid profile in nondiabetics and prediabetics in Saudi population

**DOI:** 10.1097/MD.0000000000036322

**Published:** 2023-11-24

**Authors:** Tarek Atia, Mohammad H. Abdelzaher, Somaia A. Nassar, Hoda H. Gafar, Mohammed A. M. Husseini, Abdulhadi M. Y. Kaabi, Hader I. Sakr

**Affiliations:** a Department of Medical Laboratory Sciences, College of Applied Medical Sciences, Prince Sattam Bin Abdulaziz University, Al-Kharj, Saudi Arabia; b College of Medicine, Prince Sattam Bin Abdulaziz University, Al-Kharj, Saudi Arabia; c Department of Medical Biochemistry, College of Medicine, Al-Azhar University, Assiut Branch, Assiut, Egypt; d Prince Sattam Bin Abdulaziz University Hospital, Al-Kharj, Saudi Arabia; e Department of Medical Physiology, Faculty of Medicine, Cairo University, Cairo, Egypt; f Department of Medical Physiology, Medicine program, Batterjee Medical College, Jeddah, Saudi Arabia.

**Keywords:** Fasting blood glucose, HbA1c, lipid profile, prediabetes, vitamin D deficiency

## Abstract

Vitamin D deficiency increases the risk of developing diabetes, dyslipidemia, and other chronic diseases. We aimed to investigate the relationship between vitamin D deficiency, glycemic levels, and lipid profiles in individuals with prediabetes and nondiabetes. This observational cross-sectional study was conducted on 249 adults who were divided into 2 groups based on the American Diabetes Association classification: nondiabetics and prediabetics. The serum vitamin D levels, lipid profiles, fasting blood glucose levels, hemoglobin A1c levels, fasting insulin levels, and insulin resistance (IR) were evaluated. The prevalence of vitamin D deficiency in all participants was 30.9%, and mean vitamin D levels were significantly [*P* = .0004] lower in prediabetics, who were more common in females. Furthermore, prediabetics had significantly higher serum triglycerides [*P* = .0006], and significantly lower serum high-density lipoprotein levels [*P* = .0148] than those nondiabetics. Serum cholesterol and low-density lipoprotein levels did not differ significantly between the 2 groups. 31.4% of all participants were overweight and 40.2% were obese. Furthermore, there was a strong correlation between vitamin D levels and IR and body mass indices ≥ 25 in prediabetics [r = −0.92] [*P* < .001]. Finally, vitamin D levels had a significant inverse relationship with glycemic parameters and IR, particularly in obese participants, but there was no significant relationship with lipid profile. In conclusion, vitamin D deficiency is common in females, regardless of whether they are prediabetics, but is more prevalent in prediabetics. Vitamin D deficiency is associated with high triglycerides and low high-density lipoprotein levels, but there were no significant changes in total cholesterol or low-density lipoprotein levels. Furthermore, vitamin D levels were negatively correlated with both fasting blood glucose and hemoglobin A1c levels, and its deficiency was strongly associated with IR especially in obese patients despite there being no significant correlation with blood lipids.

## 1. Introduction

Vitamin D deficiency (<20 ng/mL) is a global health problem that affects people of all ethnicities and ages. Despite Saudi Arabia sunny weather, approximately 60% of Saudis of all ages are vitamin D deficient.^[1]^ In contrast, the prevalence of acute and chronic illnesses such as diabetes, thyroid disorders, cardiovascular diseases, and cancer is increasing in this population.^[2]^ In addition to calcium metabolism and bone mineralization, vitamin D regulates cell proliferation and differentiation, protects numerous tissues from oxidative damage, plays a role in immunity and metabolism, and controls the expression of numerous genes. Vitamin D binds to the vitamin D receptors (VDR), a subclass of nuclear receptors found in nearly all of the body cells and required for the regulation of a variety of biological processes. Vitamin D deficiency has been suggested to alter the VDR, resulting in multiple organ dysfunction.^[3]^ In contrast, VDR is highly expressed in pancreatic B-cells, indicating the importance of vitamin D in regulating insulin production. Moreover, numerous studies have linked vitamin D deficiency to B-cell dysfunction and insulin resistance (IR) in type 2 diabetes.^[4,5]^ Therefore, the global increase in vitamin D deficiency may be related to the increasing prevalence of type 2 diabetes.

Prediabetes is becoming increasingly common in adults worldwide. It is estimated that one-third of US adults are at risk for type 2 diabetes (i.e., have prediabetes), as they have fasting blood glucose (FBG) or glycated hemoglobin [hemoglobin-A1c (HbA1c)] levels higher than normal, but below the diabetic threshold.^[6]^ Furthermore, it is estimated that one-fourth of the Saudi population around the age of 30 has prediabetes, and 10% develop type 2 diabetes each year.^[7]^ People with prediabetes are more likely to develop diabetic complications, such as diabetic retinopathy, nephropathy, neuropathy, and cardiovascular diseases.^[8]^ Furthermore, recent clinical trials have found that vitamin D supplements may help prediabetics avoid the development of diabetes. As a result, the early detection and intervention of prediabetes can reduce the incidence of diabetes and its complications.^[9,10]^ On the other hand, IR, a strong predictor of prediabetes, has been linked to glucose intolerance, obesity, dyslipidemia, cardiovascular disease, and cancer.^[8]^ A growing body of evidence suggests that IR is a complex metabolic disorder with multiple pathophysiological mechanisms.^[11]^ Defective insulin receptors or post-receptors compromise hormonal signal transduction mechanisms, resulting in IR. Furthermore, the IR phenomenon can be tissue- or organ-specific. Clinical studies have demonstrated a link between IR and vitamin D deficiency.^[12]^

Dyslipidemia (abnormal lipid profile levels) is a health issue worldwide in adults, especially in those with unhealthy lifestyles, obesity, and postmenopausal women. Individuals with dyslipidemia are at high risk of developing hypertension, atherosclerosis, and cardiovascular disorders.^[13]^ The relationship between vitamin D deficiency and serum lipid levels has become a research topic worldwide. Several studies have linked vitamin D deficiency to dyslipidemia, and vitamin D supplementation may improve lipid profiles in these populations.^[14,15]^ However, the link between IR and dyslipidemia has been supported by substantial evidence. IR has been linked to changes in lipid and lipoprotein metabolism, leading to atherogenic dyslipidemia and increased risk of cardiovascular disease.^[16]^ Furthermore, numerous studies have found a link between diabetes, particularly type 2, and dyslipidemia,^[17,18]^ whereas other studies have found a link between prediabetes and dyslipidemia.^[19]^

This study aimed to investigate the relationship between vitamin D levels, glycemic status, IR, and lipid profiles in a prediabetic versus nondiabetic Saudi population.

## 2. Patients and methods

The authors certify that the research was conducted in accordance with the principles of the Declaration of Helsinki and in compliance with the local regulatory requirements. The Prince Sattam bin Abdulaziz University Standing Committee of Bioethical Research [**SCBR**] approved this study (approval number: **SCBR-053-2022**) and all participants provided written informed consent to participate in the study.

An observational cross-sectional study was conducted on 249 adults who were enrolled at the University Hospital for routine checkups on general conditions such as blood glucose, lipid profile, and vitamin D levels between June 2022 and June 2023. The patients were divided into 2 groups based on the classification of the American Diabetes Association the Nondiabetic group, with FBG ≤ 5.5 mmol/L or HbA1c < 5.7%, and the prediabetes group with FBG level of > 5.5 and < 7 mmol/L or HbA1c levels of ≥ 5.7% and < 6.5%.^[20]^ The body weight, height, age, and medical history of all the patients were collected from their medical records. Furthermore, family history, education level, lifestyle factors such as physical activity and smoking status, as well as dietary factors, calcium intake, and vitamin D supplements, were reported.

The collected cases were then subjected to complete history taking, clinical examination, and laboratory tests including routine laboratory investigations such as complete blood count, and liver, renal, and thyroid function [free T3, free T4, and thyroid stimulating hormone] tests. After a 10-hour fast, blood samples were collected from all participants and divided into 2 aliquots. The first was mixed with 2 mg/mL Ethylene Diamine Tetra Acetic acid to assess complete blood count (CBC) and HbA1c levels. The second was centrifuged for 15 minutes at 3000 rpm after clotting for 30 minutes at 25°C. The sera were divided into aliquots and stored at −20°C until they were used to test 25-hydroxyvitamin D, lipid profile, thyroid function tests, fasting insulin, and FBG levels.

Serum 25-hydroxyvitamin D and fasting insulin levels were measured using a Chemiluminescent Immunoassay [Roche COBAS e411, Roche Diagnostics GmbH, Mannheim, Germany]. In addition, lipid profiles [Low-Density Lipoprotein (LDL), High-Density Lipoprotein (HDL), Triglycerides (TG), and Total Cholesterol (TC)] and FBG were also measured using enzymatic and colorimetric methods [Roche COBAS c311, Roche Diagnostics GmbH, Mannheim, Germany]. The HbA1c levels were measured using an immunoturbidimetric assay. IR was estimated using the Homeostatic Model Assessment-IR [HOMA-IR] equation. HOMA-IR, in general, has a cutoff value of 2, and a higher value indicates IR. HOMA-IR = [fasting glucose in mmol/L × fasting insulin in mIU/mL/22.5].^[21,22]^

Exclusion criteria: Patients with abnormal thyroid functions, liver and/or kidney disease, autoimmune diseases, malignancy, metabolic diseases, or hemoglobin variants were excluded from this study. Furthermore, individuals taking vitamin D supplements or lipid-lowering therapy and pregnant women were also excluded.

Statistical analysis: The results were statistically analyzed using the computer database GraphPad Prism software [version 9.1.1]. Unpaired Student’s *t* test and Pearson correlation analysis were used to compare the data. Statistical significance was set at *P* < 0.05, and the results are presented as the mean ± standard deviation.

## 3. Result

### 3.1. Population characteristics

**Figure 1.**
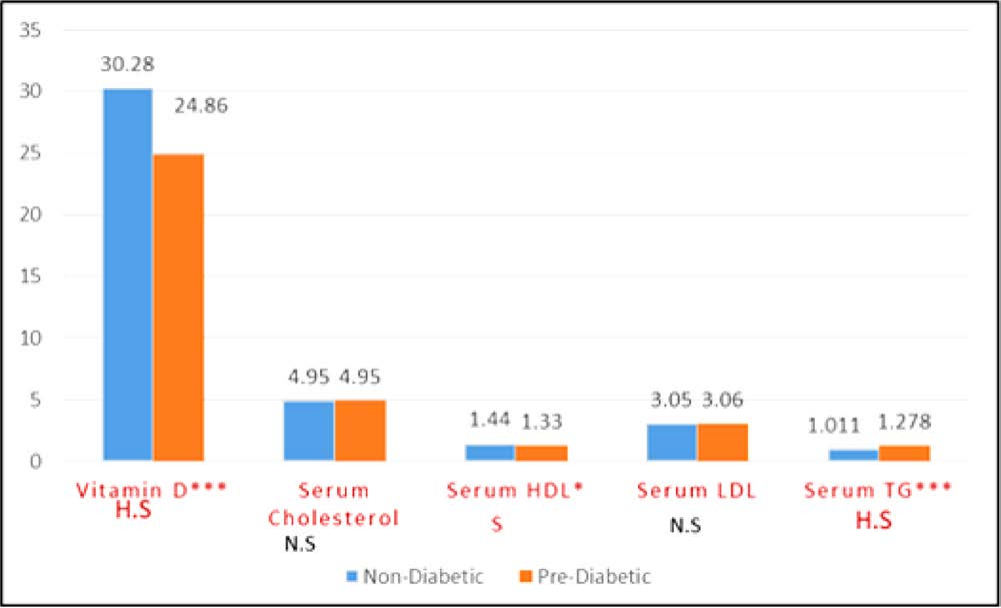
Shows that prediabetics had significantly lower vitamin D levels (24.86 ± 10.59) than nondiabetics (30.28 ± 12.51). Serum TG levels were significantly higher in prediabetics, but serum HDL levels were significantly lower. Serum cholesterol and LDL levels, on the other hand, did not differ significantly between the 2 groups. HDL = high-density lipoprotein, LDL = low-density lipoprotein, TG = triglycerides.

This study included 249 individuals: 140 women and 109 men, including 145 nondiabetics (62 men and 83 women) and 104 prediabetics (47 men and 57 women). A comparison of all the measured parameters in prediabetics and nondiabetics is summarized in Table 1 and Figure 1.

**Table 1:** A comparison of the vitamin D levels and lipid profiles in nondiabetics and prediabetes.

Parameters	Nondiabetics (n = 145)	Prediabetes (n = 104)	*P* value
Mean vitamin D levels (ng/mL) ± SD	30.28 ± 12.51	24.86 ± 10.59	.004**
Percentage of Vit. D deficiency of all participants	30.9% (n = 249) Females (64%): Males (36%)		
Percentage of Vit. D deficiency	25.5% (n = 37)	38.5% (n = 40)	
	Females: 26 (31.3%)	Females: 24 (42.1%)	
	Males: 11 (17.7%)	Males: 16 (34%)	
Serum Cholesterol (mmol/l) ± SD	4.95 ± 0.94	4.95 ± 0.88	.99*
Serum HDL (mmol/l) ± SD	1.44 ± 0.38	1.33 ± 0.33	.015**
Serum LDL (mmol/l) ± SD	3.05 ± 0.84	3.06 ± 0.85	.948*
Serum TG (mmol/l) ± SD	1.011 ± 0.46	1.28 ± 0.74	.006**

Table 1 demonstrates that the mean vitamin D levels in prediabetes (24.86 ± 10.59) were significantly lower (*P* < .0004) than in nondiabetics (30.28 ± 12.51). The serum TG level was significantly higher (*P* < .0006), but the serum HDL level was significantly lower (*P* < .0148) in prediabetes compared to non-diabetics. In contrast, the serum cholesterol and LDL levels did not differ significantly between the 2 groups.

LDL = low-density lipoprotein, *P* value = probability value, SD = standard deviation, TG = triglycerides.

* = Non-significant (NS)

** = significant values (S).

### 3.2. Vitamin D status in the study population:

Vitamin D deficiency [<20 ng/mL] was found in 30.9% of all participants, with females more affected [64%] than males [36%]. The mean vitamin D level was significantly lower [*P* = .0004] in the prediabetics. Furthermore, the percentage of vitamin D deficiency in prediabetics was 38.5% [40 cases: 24 females and 16 males], significantly higher than that in nondiabetics 25.5% [37 cases: 26 females and 11 males].

### 3.3. Lipid profile status in the study population

Furthermore, the serum TG level was significantly higher [*P* = .0006], but the serum HDL level was significantly lower [*P* = .0148] in prediabetics than in nondiabetics. However, the serum cholesterol and LDL levels did not differ significantly between the 2 groups (Table 1).

### 3.4. Correlation between vitamin D levels and the tested parameters

There was a significant negative correlation between vitamin D levels and both HbA1c and FBG levels [r = −0.21, r = −0.23] [*P* = .008, *P* = .0003] (Table 2). Furthermore, this significant negative correlation was found in both males [r = −0.31, r = −0.25] [*P* = .001, *P* = .009] and females [r = −0.2, r = −0.21] [*P* = .017, *P* = .012] (Table 3). In contrast, no significant relationship was found between vitamin D levels and lipid profile parameters [cholesterol, LDL, HDL, and TG levels] (Table 2).

**Table 2:** The correlations between vitamin D levels and FBG, HbA1c, and lipid profiles in all participants.

Parameters	Pearson r	*P* value
Vitamin D & HbA1c	−0.21	.008**
Vitamin D & FBG	−0.23	.003**
Vitamin D & Cholesterol	−0.01	.851*
Vitamin D & HDL	0.12	.059*
Vitamin D & LDL	−0.05	.455*
Vitamin D &TG	−0.07	.257*

Table 2 shows the correlation between vitamin D levels and the tested parameters (HbA1c, FBG, cholesterol, HDL, LDL, and TG levels) in all participants. There was a significant negative correlation between vitamin D, FBG, and HbA1c levels. There was no significant relationship between the vitamin D levels and lipid profiles (cholesterol, LDL, HDL, and TG).

FBG = fasting blood glucose, LDL = low-density lipoprotein, TG = triglycerides.

* = Non-significant correlation

** = significant negative correlation.

**Table 3:** Correlation analysis between vitamin D levels and FBG and HbA1c in all participants based on gender.

	Parameters	Number of cases	Pearson r	*P* value
Females	Vitamin D & FBG	140	−0.2	.017**
Males	Vitamin D & FBG	109	−0.31	.001**
Females	Vitamin D & HbA1c	140	−0.21	.012**
Males	Vitamin D & HbA1c	109	−0.25	.009**

Table 3 shows the correlation between vitamin D levels (ng/mL) and HbA1c% and FBG (mmol/l) in all participants, where both males and females had a significant negative correlation between vitamin D levels and FBG and HbA1c levels.

FBG = fasting blood glucose.

** = Significant negative correlation.

### 3.5. Body mass index (BMI) and fasting insulin levels in prediabetes and non-diabetes

According to our findings, the mean BMI of male participants [28.8 ± 5.6] was non-significantly higher than that of female participants [28.4 ± 5.2]. Furthermore, 31.4% of all participants were overweight [BMI < 30 and ≥ 25], and 40.2% were obese [BMI ≥ 30]. On the other hand, 77.9% of prediabetics had a BMI of ≥ 25, which was slightly higher than in nondiabetics [66.2% with a BMI of ≥ 25].

The mean fasting insulin level in prediabetics [16.3 ± 11.1 mIU/mL] was significantly higher than in nondiabetics [8.7 ± 6.1 mIU/mL] [*P* < .0001]. When we examined IR in both groups and its relationship to glycemic status, we found that 85.2% of prediabetics with BMI ≥ 25 had HOMA -IR > 2, which was significantly higher than the [27%] nondiabetics [*P* < .0001].

Furthermore, 89.4% of prediabetics had IR, which was significantly higher than the 19.3% of nondiabetics [*P* < .0001]. Despite having a BMI of ≥ 25, 27% of the nondiabetics had HOMA-IR > 2 and normal FBG and HbA1c (Table 4).

**Table 4:** Comparison between BMI and insulin resistance status in both nondiabetics and prediabetics.

	Prediabetics (n = 104)	Nondiabetics (n = 145)
BMI < 25	22.1% (23 patients)	33.8% (49)
BMI ≥ 25	77.9% (81)	66.2% (96)
Fasting insulin (mIU/mL) Mean ± SD	16.3 ± 11.1 mIU/mL (*P* < .0001	8.7 ± 6.1 mIU/mL
HOMA-IR < 2	10.6% (11)	80.7% (117)
HOMA-IR > 2	89.4% (93)	19.3% (28)
HOMA-IR > 2 & BMI ≥ 25	85.2% (69)	27% (26)

BMI = body mass index, HOMA-IR = Homeostatic Model Assessment-Insulin Resistance.

### 3.6. Correlation between fasting insulin levels, IR, and vitamin D levels

Furthermore, there was a significant negative correlation between vitamin D levels and fasting insulin levels among prediabetics [r = −0.85] [*P* < .001], but not among nondiabetics [r = −0.15] [*P* = .07]. When investigating the relationship between vitamin D status and IR according to BMI, we noticed that there was a strong correlation between vitamin D levels and cases of IR [HOMA-IR > 2 and BMI ≥ 25] in prediabetics [r = −0.92; *P* < .001], and a mild correlation in nondiabetics [r = −0.19; *P* = .022]. In contrast, no correlation was found between cases in either group with HOMA-IR > 2 and BMI < 25 [r = −0.17; *P* = .63] and [r = −0.11; *P* = .67] (Table 5).

**Table 5:** Correlation analysis between vitamin D levels and insulin resistance according to BMI in both Nondiabetic and prediabetic groups.

	Prediabetics (104)	Nondiabetics (145)
Correlation between fasting insulin levels and vitamin D levels	Pearson r (r = −0.85)	Pearson r (r = −0.15)
	*P* value (*P* < .001)	*P* value (*P* = .07)
Correlation between patients with HOMA-IR > 2 values with BMI ≥ 25 and their vitamin D levels	Pearson r (r = −0.92)	Pearson r (r = −0.19)
	*P* value (*P* < .001)	*P* value (*P* = .022)
Correlation between cases with HOMA-IR > 2 values with BMI < 25 and vitamin D levels	Pearson r (r = −0.17)	Pearson r (r = −0.11)
	*P* value (*P* = .63)	*P* value (*P* = .67)

BMI = body mass index, HOMA-IR = Homeostatic Model Assessment-Insulin Resistance.

## 4. Discussion

The prevalence of vitamin D deficiency, prediabetes, and diabetes is increasing worldwide, including in Saudi Arabia. Vitamin D deficiency is a risk factor for diabetes and dyslipidemia.^[14]^ Our study revealed that 30.9% of participants were vitamin D deficient, with females being more affected, which was consistent with a previous study among Saudis.^[23]^ AlFaris et al^[24]^ stated that vitamin D deficiency is a common health problem in Middle Eastern countries, with females being more affected. However, traditional female clothing in Saudi Arabia, diet, education level, and lifestyle may be predisposing factors for vitamin D deficiency.^[25]^

Furthermore, there was a significant [*P* = .0004] reduction in the main levels of vitamin D in pre-diabetics compared to non-diabetics. However, several studies have investigated the vitamin D levels in patients with diabetes and prediabetes. Gao et al^[26]^ found a significant relationship between low vitamin D levels and increased risk of prediabetes. In addition, Bhatt et al^[27]^ reported that lower vitamin D levels are associated with higher FBG levels in Asian Indian women with prediabetes. Furthermore, the National Health and Nutrition Examination Survey [2001–2006] found that vitamin D levels in patients with prediabetes and diabetes were significantly lower than those in individuals with normal blood sugar levels.^[25]^ In another study, vitamin D deficiency was associated with poor glycemic control in patients with type 2 diabetes,^[28]^ implying that vitamin D deficiency contributes to the onset and progression of IR and type 2 diabetes.^[29]^

However, in our study, we found a significant negative correlation between vitamin D levels and both HbA1c and FBG levels [*P* = .008 and *P* = .0003 respectively]. This study found that vitamin D deficiency affected the glycemic parameters in both diabetic and prediabetic patients. In addition, other studies have found an inverse relationship between vitamin D levels and both FBG and HbA1c levels in study participants.^[30]^ Tang et al^[31]^ reported that vitamin D supplementation significantly reduced FBG levels in nondiabetic patients. However, another study reported that vitamin D supplementation did not affect FBG control, IR, or type 2 diabetes in nondiabetics.^[32,33]^ In contrast, IR was improved in individuals with type 2 diabetes and vitamin D levels ≥ 30 ng/mL.^[34]^ Vitamin D improves insulin secretion by stimulating the VDR in pancreatic beta cells.^[35]^ In addition, vitamin D can indirectly improve insulin secretion by normalizing extracellular calcium levels and maintaining normal intracellular calcium influx.^[36]^ Furthermore, vitamin D can improve insulin sensitivity by increasing the expression of insulin receptors and promoting the expression of peroxisome proliferator-activated receptor [PPAR], a nuclear receptor involved in lipid and glucose metabolism.^[37]^

In the current study, we investigated IR and vitamin D deficiency in both groups based on BMI and their relationship with glycemic status. Our results revealed a significant relationship between IR, vitamin D deficiency, and BMI ≥ 25 in both groups, with prediabetics having a higher significance than nondiabetics. However, the relationship between vitamin D deficiency and IR, metabolic syndrome, diabetes, and impaired beta cell function, has been established. This correlation varies depending on the race of the population.^[38]^ Furthermore, vitamin D deficiency has been linked to prediabetes, being overweight, or being obese.^[39]^ Moreover, in a large meta-analysis, vitamin D deficiency was linked to increased BMI in diabetics and nondiabetics.^[40]^ Taken together, our findings are consistent with those of previous studies on the relationship between vitamin D deficiency, IR, and BMI in prediabetics and nondiabetics.^[39,40]^

Our results showed that the prevalence of dyslipidemia was more common in patients with prediabetes. However, prediabetics had significantly lower HDL [*P* = .014] and higher TG [*P* = .0006] levels than nondiabetics. In contrast, the serum cholesterol and LDL levels in our study showed no significant differences between prediabetics and nondiabetics. Furthermore, no correlation was found between vitamin D levels and lipid profile fractions [TC, TG, LDL, and HDL] in any of the participants. This result was consistent with previous studies.^[14,41]^ In addition, Wang et al^[42]^ reported that a decrease in the level of vitamin D was associated with higher levels of TG and lower levels of HDL in males than in females. Ponda et al^[43]^ reported that vitamin D deficiency [<20 ng/mL] is associated with an abnormal lipid profile, with lower TC, TG, and LDL levels, but higher HDL levels.

Vitamin D deficiency may result in IR, which could affect lipoprotein metabolism resulting in increased TG and reduced HDL levels.^[36]^ In contrast, studies have shown that vitamin D can improve dyslipidemia by stimulating calcium absorption in the small intestine, thus lowering both TG production and saturated fatty acids absorption.^[44]^ Furthermore, VDR overexpression reduces lipid catabolism, promotes lipogenesis pathways, and improves adipocyte differentiation and proliferation.^[45]^

Moreover, improving vitamin D levels to normal levels [≥30 ng/mL] failed to improve TG and LDL levels.^[46]^ Furthermore, AlAnouti et al^[47]^ conducted a comprehensive systematic review and meta-analysis of the effects of vitamin D supplementation on dyslipidemia in patients with metabolic syndrome. The study concluded that vitamin D supplementation did not affect dyslipidemia in the study population. In contrast, another study reported that vitamin D supplementation can improve type 2 diabetes and its complications including dyslipidemia.^[48]^ However, whether vitamin D deficiency affects lipid profile fractions is controversial, because multiple factors, such as age, sex, physical activity, seasonal variation, and diabetes may play a role. In addition, lipoprotein and plasma lipid levels vary with the season, with higher concentrations of TC and LDL observed in winter, and lower concentrations in summer.^[49]^

## 5. Conclusion

Vitamin D deficiency is common in females, regardless of whether they are prediabetics, but it is more prevalent in patients with prediabetes. Vitamin D deficiency was associated with high TG and low HDL levels, but there were no significant changes in TC or LDL levels. Furthermore, vitamin D levels are negatively correlated with both FBG and HbA1c levels, and its deficiency is strongly associated with IR, especially in obese patients, despite the lack of significant correlation with blood lipids.

Limitation of the study: The study is limited by its small sample size.

## Author contributions

**Conceptualization:** Tarek Atia, Hader Sakr.

**Data curation:** Mohammad Abdelzaher, Hoda H Gafar, Mohammed Husseini.

**Formal analysis:** Somaia Nassar, Mohammed Husseini.

**Investigation:** Tarek Atia, Hoda H Gafar, Abdulhadi Kaabi, Hader Sakr.

**Methodology:** Somaia Nassar, Hoda H Gafar, Mohammed Husseini, Abdulhadi Kaabi.

**Project administration:** Tarek Atia, Somaia Nassar, Abdulhadi Kaabi, Hader Sakr.

**Resources:** Hader Sakr.

**Software:** Hoda H Gafar, Abdulhadi Kaabi.

**Supervision:** Hader Sakr.

**Validation:** Tarek Atia, Somaia Nassar, Hader Sakr.

**Visualization:** Tarek Atia, Mohammed Husseini, Hader Sakr.

**Writing – original draft:** Tarek Atia.

**Writing – review & editing:** Tarek Atia, Mohammad Abdelzaher, Somaia Nassar.

## References

[1] Al-Alyani H Al-Turki HA Al-Essa ON. Vitamin D deficiency in Saudi Arabians: a reality or simply hype: a meta-analysis [2008–2015]. J Family Community Med. 2018;25:1–4. 29386955 10.4103/jfcm.JFCM_73_17 PMC5774037

[2] Singh P Kumar M Al Khodor S. Vitamin D deficiency in the gulf cooperation council: exploring the triad of genetic predisposition, the gut microbiome and the immune system. Front Immunol. 2019;10:1042. 31134092 10.3389/fimmu.2019.01042 PMC6524467

[3] Bikle D. Vitamin D: production, metabolism, and mechanisms of action. [Updated 2017 Aug 11]. In: Feingold KR Anawalt B Boyce A. editors. Endotext. South Dartmouth [MA]: MDText.com, Inc.; 2000. Available from: https://www.ncbi.nlm.nih.gov/books/NBK278935/

[4] Atia T Iqbal MZ Fathy AH. Vitamin D supplementation could enhance the effectiveness of glibenclamide in treating diabetes and preventing diabetic nephropathy: a Biochemical, Histological and Immunohistochemical Study. J Evid Based Integr Med. 2022;27:2515690X–221116403. 10.1177/2515690X221116403 PMC9393666 35942573

[5] Wu J Atkins A Downes M. Vitamin D in diabetes: uncovering the sunshine hormone’s role in glucose metabolism and beyond. Nutrients. 2023;15:1997. 37111216 10.3390/nu15081997 PMC10142687

[6] Davidson KW Barry MJ Mangione CM. Screening for prediabetes and type 2 diabetes: us preventive services task force recommendation statement. JAMA. 2021;326:736–43. 34427594 10.1001/jama.2021.12531

[7] Alateeq MA Aljohani M Kinani SS. The prediabetes outcome at national guard primary health care centers in Riyadh, Saudi Arabia: retrospective chart review. Cureus. 2020;12:e10227. 33042670 10.7759/cureus.10227 PMC7536105

[8] Al Amri T Bahijri S Al-Raddadi R. The association between prediabetes and dyslipidemia among attendants of primary care health centers in Jeddah, Saudi Arabia. Diabetes Metab Syndr Obes. 2019;12:2735–43. 31920353 10.2147/DMSO.S233717 PMC6935271

[9] Pittas AG Jorde R Kawahara T. Vitamin D supplementation for prevention of type 2 diabetes mellitus: to D or not to D? J Clin Endocrinol Metab. 2020;105:3721–33. 32844212 10.1210/clinem/dgaa594 PMC7571449

[10] Amrein K Scherkl M Hoffmann M. Vitamin D deficiency 20: an update on the current status worldwide. Eur J Clin Nutr. 2020;74:1498–513. 31959942 10.1038/s41430-020-0558-y PMC7091696

[11] Li M Chi X Wang Y. Trends in insulin resistance: insights into mechanisms and therapeutic strategy. Signal Transduct Target Ther. 2022;7:216. 35794109 10.1038/s41392-022-01073-0 PMC9259665

[12] Trimarco V Manzi MV Mancusi C. Insulin resistance and vitamin D deficiency: a link beyond the appearances. Front Cardiovasc Med. 2022;9:859793. 35369303 10.3389/fcvm.2022.859793 PMC8968037

[13] Lokpo SY Laryea R Osei-Yeboah J. The pattern of dyslipidaemia and factors associated with elevated levels of non-HDL-cholesterol among patients with type 2 diabetes mellitus in the Ho municipality: a cross sectional study. Heliyon. 2022;8:e10279. 36046539 10.1016/j.heliyon.2022.e10279 PMC9421188

[14] AlQuaiz AM Kazi A Youssef RM. Association between standardized vitamin D and dyslipidemia: a community-based study in Riyadh, Saudi Arabia. Environ Health Prev Med. 2020;25:4. 31941477 10.1186/s12199-019-0841-5 PMC6964076

[15] Surdu AM Pînzariu O Ciobanu DM. Vitamin D and its role in the lipid metabolism and the development of atherosclerosis. Biomedicines. 2021;9:172. 33572397 10.3390/biomedicines9020172 PMC7916166

[16] Bjornstad P Eckel RH. Pathogenesis of lipid disorders in insulin resistance: a brief review. Curr Diab Rep. 2018;18:127. 30328521 10.1007/s11892-018-1101-6 PMC6428207

[17] Hirano T. Pathophysiology of diabetic dyslipidemia. J Atheroscler Thromb. 2018;25:771–82. 29998913 10.5551/jat.RV17023 PMC6143775

[18] Banach M Surma S Reiner Z. Personalized management of dyslipidemias in patients with diabetes—it is time for a new approach (2022). Cardiovasc Diabetol. 2022;21:263. 36443827 10.1186/s12933-022-01684-5 PMC9706947

[19] Sundharan S Ramchandran S Jatale R. Dyslipidemia in prediabetes population: a retrospective study of 91780 cases. Int J Health Sci Res. 2022;12:132–9.

[20] Li G Han L Wang Y. Evaluation of ADA HbA1c criteria in the diagnosis of pre-diabetes and diabetes in a population of Chinese adolescents and young adults at high risk for diabetes: a cross-sectional study. BMJ Open. 2018;8:e020665. 10.1136/bmjopen-2017-020665 PMC6089273 30093511

[21] Katz A Nambi SS Mather K. Quantitative insulin sensitivity check index: a simple, accurate method for assessing insulin sensitivity in humans. J Clin Endocrinol Metab. 2000;85:2402–10. 10902785 10.1210/jcem.85.7.6661

[22] Wallace TM Levy JC Matthews DR. Use and abuse of HOMA modeling. Diabetes Care. 2004;27:1487–95. 15161807 10.2337/diacare.27.6.1487

[23] Alsuwadia AO Farag YM Al Sayyari AA. Prevalence of vitamin D deficiency in Saudi adults. Saudi Med J. 2013;34:814–8. 23974452

[24] AlFaris NA AlKehayez NM AlMushawah FI. Vitamin D deficiency and associated risk factors in women from Riyadh, Saudi Arabia. Sci Rep. 2019;9:20371. 31889122 10.1038/s41598-019-56830-z PMC6937288

[25] Salih YA Rasool MT Ahmed IH. Impact of vitamin D level on glycemic control in diabetes mellitus type 2 in Duhok. Ann Med Surg (2012). 2021;64:102208. 10.1016/j.amsu.2021.102208 PMC7988274 33786167

[26] Gao Y Zheng T Ran X. Vitamin D and incidence of prediabetes or type 2 diabetes: a four-year follow-up community-based study. Dis Markers. 2018;2018:1–8. 10.1155/2018/1926308 PMC5878872 29743959

[27] Bhatt SP Misra A Gulati S. Lower vitamin D levels are associated with higher blood glucose levels in Asian Indian women with pre-diabetes: a population-based cross-sectional study in North India. BMJ Open Diabetes Research and Care 2018;6:e000501. 10.1136/bmjdrc-2017-000501 PMC6014203 29942523

[28] Al Dossari KK Ahmad G Aljowair A. Association of vitamin D with glycemic control in Saudi patients with type 2 diabetes: a retrospective chart review study in an emerging university hospital. J Clin Lab Anal. 2020;34:e23048. 31568604 10.1002/jcla.23048 PMC7031596

[29] Vondra K Hampl R. Vitamin D and new insights into pathophysiology of type 2 diabetes. Horm Mol Biol Clin Investig. 2021;42:203–8. 10.1515/hmbci-2020-0055 33655734

[30] Alharazy S Aliss E Lanham-New S. Association between vitamin D and glycaemic parameters in a multi-ethnic cohort of postmenopausal women with type 2 diabetes in Saudi Arabia. BMC Endocr Disord. 2021;21:162. 34380489 10.1186/s12902-021-00825-3 PMC8359582

[31] Tang H Li D Li Y. Effects of vitamin D supplementation on glucose and insulin homeostasis and incident diabetes among nondiabetic adults: a meta-analysis of randomized controlled trials. Int J Endocrinol. 2018;2018:7908764. 30627160 10.1155/2018/7908764 PMC6304827

[32] Mousa A Naderpoor N de Courten MP. Vitamin D supplementation has no effect on insulin sensitivity or secretion in vitamin D-deficient, overweight or obese adults: a randomized placebo-controlled trial. Am J Clin Nutr. 2017;105:1372–81. 28490514 10.3945/ajcn.117.152736

[33] Fondjo LA Owiredu WKBA Sakyi SA. Vitamin D status and its association with insulin resistance among type 2 diabetics: a case-control study in Ghana. PLoS One. 2017;12:e0175388. 28423063 10.1371/journal.pone.0175388 PMC5396912

[34] Wang L Liu X Hou J. Serum vitamin D affected type 2 diabetes though altering lipid profile and modified the effects of testosterone on diabetes status. Nutrients. 2020;13:90. 33396618 10.3390/nu13010090 PMC7823697

[35] He S Yu S Zhou Z. Effect of vitamin D supplementation on fasting plasma glucose, insulin resistance and prevention of type 2 diabetes mellitus in nondiabetics: a systematic review and meta-analysis. Biomed Rep. 2018;8:475–84. 29725526 10.3892/br.2018.1074 PMC5920274

[36] Contreras-Bolívar V García-Fontana B García-Fontana C. Mechanisms involved in the relationship between vitamin D and insulin resistance: impact on clinical practice. Nutrients. 2021;13:3491. 34684492 10.3390/nu13103491 PMC8539968

[37] Muñoz-Garach A García-Fontana B Muñoz-Torres M. Vitamin D status, calcium intake and risk of developing type 2 diabetes: an unresolved issue. Nutrients. 2019;11:642. 30884820 10.3390/nu11030642 PMC6471926

[38] Xu Z Gong R Luo G. Association between vitamin D3 levels and insulin resistance: a large sample cross-sectional study. Sci Rep. 2022;12:119. 34997087 10.1038/s41598-021-04109-7 PMC8741779

[39] Bhatt SP Misra A Pandey RM. Vitamin D supplementation in overweight/obese Asian Indian women with prediabetes reduces glycemic measures and truncal subcutaneous fat: a 78 weeks randomized placebo-controlled trial [PREVENT-WIN Trial]. Sci Rep. 2020;10:220. 31937856 10.1038/s41598-019-56904-y PMC6959323

[40] Rafiq S Jeppesen PB. Body mass index, vitamin D, and type 2 diabetes: a systematic review and meta-analysis. Nutrients. 2018;10:1182. 30154381 10.3390/nu10091182 PMC6164132

[41] Hoseini R Damirchi A Babaei P. Vitamin D increases PPARγ expression and promotes beneficial effects of physical activity in metabolic syndrome. Nutrition. 2017;36:54–9. 28336108 10.1016/j.nut.2016.06.010

[42] Wang Y Si S Liu J. The associations of serum lipids with vitamin D status. PLoS One. 2016;11:e0165157. 27768777 10.1371/journal.pone.0165157 PMC5074586

[43] Ponda MP Huang X Odeh MA. Vitamin D may not improve lipid levels: a serial clinical laboratory data study. Circulation. 2012;126:270–7. 22718799 10.1161/CIRCULATIONAHA.111.077875 PMC3713625

[44] Park CY Han SN. The role of vitamin D in adipose tissue biology: adipocyte differentiation, energy metabolism, and inflammation. J Lipid Atheroscler. 2021;10:130–44. 34095008 10.12997/jla.2021.10.2.130 PMC8159757

[45] Szymczak-Pajor I Drzewoski J Sliwinska A. The molecular mechanisms by which vitamin D prevents insulin resistance and associated disorders. Int J Mol Sci. 2020;21:6644. 32932777 10.3390/ijms21186644 PMC7554927

[46] Rashidi H Ghaderian SB Moradi L. The effect of vitamin D3 on improving lipid profile, fasting glucose and insulin resistance in polycystic ovary syndrome women with vitamin D deficiency. Middle East Fertil Soc J. 2018;23:178–83.

[47] AlAnouti F Abboud M Papandreou D. Effects of vitamin D supplementation on lipid profile in adults with the metabolic syndrome: a systematic review and meta-analysis of randomized controlled trials. Nutrients 2020;12:3352. 33143204 10.3390/nu12113352 PMC7692169

[48] Tabatabaei-Malazy O Peimani M Mohsen S.. Therapeutic effects of dietary antioxidative supplements on the management of type 2 diabetes and its complications; umbrella review of observational/trials meta-analysis studies. J Diabetes Metab Disord. 2022;21:1833–59. 36404841 10.1007/s40200-022-01069-1 PMC9672207

[49] Skutecki R Cymes I Dragańska E. Are the levels of lipid parameters associated with biometeorological conditions? Int J Environ Res Public Health. 2019;16:4636. 31766531 10.3390/ijerph16234636 PMC6926572

